# Hepatogenic and neurogenic differentiation of bone marrow mesenchymal stem cells from abattoir-derived bovine fetuses

**DOI:** 10.1186/1746-6148-10-154

**Published:** 2014-07-10

**Authors:** Fernando Dueñas, Víctor Becerra, Yennifer Cortes, Sonia Vidal, Leonardo Sáenz, Jaime Palomino, Mónica De los Reyes, Oscar A Peralta

**Affiliations:** 1 Departamento de Fomento de la Producción Animal, Facultad de Ciencias Veterinarias y Pecuarias, Universidad de Chile, Santiago, Chile; 2 Departamento de Ciencias Biológicas Animales, Facultad de Ciencias Veterinarias y Pecuarias, Universidad de Chile, Santiago, Chile; 3 Department of Biomedical Sciences and Pathobiology, Virginia-Maryland Regional College of Veterinary Medicine, Virginia Polytechnic Institute and State University, Blacksburg, VA 24061-0442, USA

**Keywords:** Bovine fetuses, Mesenchymal stem cell, Differentiation potential, Hepatocyte-like cell, Neuron-like cell

## Abstract

**Background:**

Mesenchymal stem cells (MSC) are multipotent progenitor cells characterized by their ability to both self-renew and differentiate into tissues of mesodermal origin. The plasticity or transdifferentiation potential of MSC is not limited to mesodermal derivatives, since under appropriate cell culture conditions and stimulation by bioactive factors, MSC have also been differentiated into endodermal (hepatocytes) and neuroectodermal (neurons) cells. The potential of MSC for hepatogenic and neurogenic differentiation has been well documented in different animal models; however, few reports are currently available on large animal models. In the present study we sought to characterize the hepatogenic and neurogenic differentiation and multipotent potential of bovine MSC (bMSC) isolated from bone marrow (BM) of abattoir-derived fetuses.

**Results:**

Plastic-adherent bMSC isolated from fetal BM maintained a fibroblast-like morphology under monolayer culture conditions. Flow cytometric analysis demonstrated that bMSC populations were positive for MSC markers CD29 and CD73 and pluripotency markers OCT4 and NANOG; whereas, were negative for hematopoietic markers CD34 and CD45. Levels of mRNA of hepatic genes α-fetoprotein (*AFP*), albumin (*ALB*), alpha1 antitrypsin (*α1AT*), connexin 32 (*CNX32*), tyrosine aminotransferase (*TAT*) and cytochrome P450 (*CYP3A4*) were up-regulated in bMSC during a 28-Day period of hepatogenic differentiation. Functional analyses in differentiated bMSC cultures evidenced an increase (P < 0.05) in albumin and urea production and glycogen storage. bMSC cultured under neurogenic conditions expressed NESTIN and MAP2 proteins at 24 h of culture; whereas, at 144 h also expressed TRKA and PrP^C^. Levels of *MAP2* and *TRKA* mRNA were up-regulated at the end of the differentiation period. Conversely, bMSC expressed lower levels of *NANOG* mRNA during both hepatogenic and neurogenic differentiation processes.

**Conclusion:**

The expression patterns of linage-specific markers and the production of functional metabolites support the potential for hepatogenic and neurogenic differentiation of bMSC isolated from BM of abattoir-derived fetuses. The simplicity of isolation and the potential to differentiate into a wide variety of cell lineages lays the foundation for bMSC as an interesting alternative for investigation in MSC biology and eventual applications for regenerative therapy in veterinary medicine.

## Background

Mesenchymal stem cells (MSC) are multipotent progenitor cells characterized by their ability to both self-renew and differentiate into tissues of mesodermal origin (osteoblasts, adipocytes, chondrocytes and myocytes) [1]. Despite several tissues have been explored for the isolation of MSC including adipose, umbilical and placental; animal bone marrow (BM) is the most common source of MSC for clinical and research uses. MSC are directly isolated from bone marrow aspirates based on their ability to adhere to plastic when plated in monolayer culture and thereafter replicate *ex vivo* to form a phenotypically homogeneous population of cells [2]. Plastic adherence under standard culture conditions is one of the criteria for defining MSC by the International Society for Cellular Therapy (ISCT). Other requirements include trilineage differentiation potential and expression of MSC surface antigens markers CD105 (endoglin), CD73 (ecto-5’-nucleotidase) and CD90 (Thy-1), and lack of expression of hematopoietic markers CD45 (protein tyrosine phosphatase, receptor type, C), CD34 (CD34 molecule) and CD14 (CD14 molecule) [3].

Despite the wide relevance of the bovine experimental model in both *in vivo* and *in vitro* experiments, limited information regarding bovine MSC (bMSC) is available. Similarities in organ size and physiology with humans and a longer life span in comparison with traditional laboratory animal models support the use of large animal models for long-term experiments in regenerative medicine [4,5]. Development of a bMSC model would be invaluable for testing the efficiency and safety of these cells for future cell therapies and for the creation of human disease models. Moreover, cattle can give advantages for clinical applications of MSC to human and veterinary medicine especially in musculoskeletal disorders [6-8].

Previous studies reported isolation and mesenchymal differentiation of bMSC from calf BM [7,9] and bovine umbilical cord [10,11]. We have reported the isolation and mesenchymal multilineage differentiation of bMSC derived from BM of abattoir-derived bovine fetuses [12]. Studies performed on human MSC isolated from fetal BM have shown that these cells possess higher proliferative capacity, trilineage differentiation potential and lower immunogenicity compared to MSC from umbilical cord, adult BM or adipose tissue [13]. Moreover, human fetal MSC isolated from BM had higher proliferative and osteogenic capacity than MSC derived from other ontological and anatomical origins, suggesting that they are superior candidates for bone tissue engineering [13,14]. The simplicity of isolation and the potential to differentiate into several cell types lays the foundation for fetal bMSC, as an interesting source for investigation of stem cell biology. Moreover, the development of large animal experimental models including cattle may open alternative strategies for investigating MSC physiology and eventual applications for regenerative therapy in human and veterinary medicine. As an example, recently it has been proposed the potential application of various stem/progenitor cells including mammary stem cells for the repair of post-mastitis structural defects in dairy animals [15,16].

The plasticity or transdifferentiation potential of MSC is not limited to mesenchymal derivatives, since under appropriate cell culture conditions and stimulation by certain exogenous or endogenous bioactive factors, MSC have also been differentiated into endodermal (hepatocytes) and neuroectodermal (neurons) cells [17,18]. The potential for hepatogenic differentiation of MSC has been evaluated by measuring the expression of endodermal or hepatocyte markers including α-fetoprotein (AFP), albumin (ALB), alpha1 antitrypsin (α1AT), connexin 32 (CNX32), tyrosine aminotransferase (TAT) and cytochrome P450 (CYP3A4) [19]. The functional capacity has also been assessed by determining urea production and glycogen storage [18]. Moreover, a large body of evidence has established the neural differentiation potential of MSC derived from BM. Treatment of BM-MSC with different molecules and growth factors induced rapid morphological changes that are typical of neural cells together with the expression of neural markers such as neuroepithelial stem cell intermediate filament (NESTIN), microtubule associated protein 2 (MAP2), nerve growth factor (NGF), tropomyosin-related kinase A (TRKA) and cellular prion protein (PrP^C^) [20-22]. Thus, the potential for isolation and differentiation into hepatocyte- and neuron-like cells, as well as their capacity for transplantation, suggests that MSC represent an attractive therapeutic candidate for treating degenerative diseases.

In the present study, we used abattoir-derived bovine fetuses as an available and plentiful source of BM with the aim to obtain an abundant supply of bMSC for *in vitro* differentiation experiments. Our main objective was to characterize the hepatogenic and neurogenic differentiation potential of bMSC isolated from abattoir-derived bovine fetuses by gene expression and functional analyses.

## Results

### Mesenchymal cell surface marker and multipotent profile of BM-bMSC from abattoir-derived fetuses

**Figure 1:**
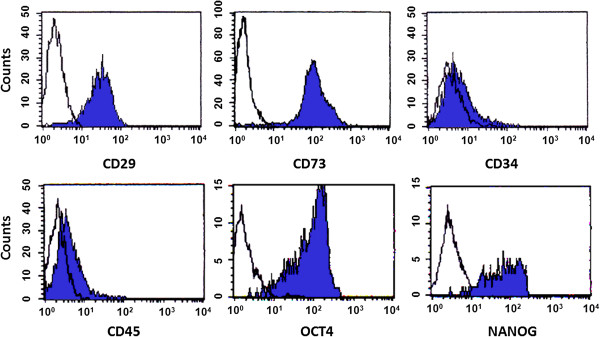
**Flow cytometric analysis of bMSC isolated by plastic adherence from BM of abattoir-derived fetuses.** Higher percentage (P < 0.05) of bMSC (shaded region) was positive for MSC surface antigens CD29, CD73 and negative for hematopoietic markers CD34 and CD45. Similarly, the majority of MSC expressed pluripotent genes OCT4 and NANOG. Open region in each panel represent the control.

Fetal bMSC were isolated from BM based on the capacity for plastic attachment under standard culture conditions that included DMEM media supplemented with 10% FBS. After 5 to 6 days of culture, colonies of fibroblast-like cells were visualized attached to plastic culture flasks. Isolated cells were cultured for several weeks in monolayer and used for differentiation experiments after 4 to 5 passages. Flow cytometric analysis of bMSC demonstrated that higher (P < 0.05) percentage of these cells were positive for MSC markers CD29 (76.3%) and CD73 (96.8%) and pluripotency markers OCT4 (94.6%) and NANOG (88.4%) (Figure 1). In comparison, a higher (P < 0.05) percentage of cells was negative for hematopoietic markers CD34 and CD45 (93.4% and 95.6%, respectively).

### Gene expression profile in bMSC during hepatogenic differentiation

**Figure 2:**
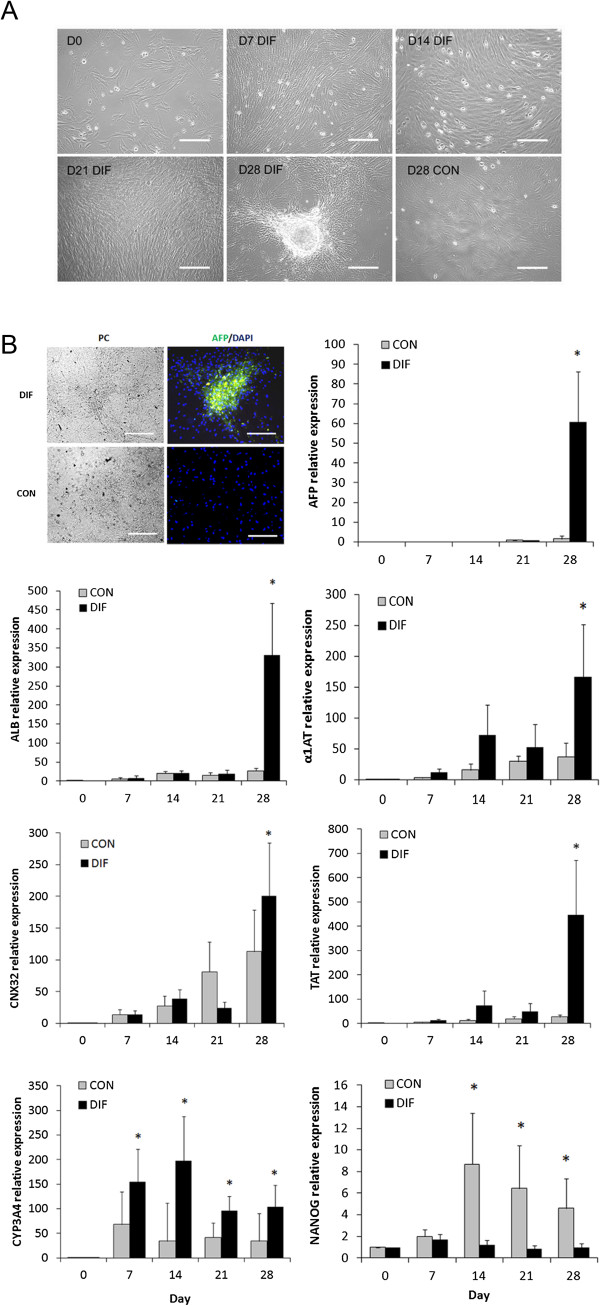
**Hepatocyte and multipotent gene expression profile in bMSC under hepatogenic conditions during a 28-Day *in vitro *culture period. (A)** bMSC were isolated by plastic adherence from bovine fetal BM and cultured under hepatogenic or control conditions. Culture of fetal bMSC under hepatogenic conditions stimulated cell replication at earlier stages and induced formation of polygonal cell morphology and cell aggregates at Day 28 of culture. **(B)** AFP protein was immunodetected in cell aggregates in differentiated (DIF) but not in control (CON) bMSC cultures at Day 28. AFP mRNA levels increased (P < 0.05) in differentiated bMSC at Day 28 of culture. Similarly, ALB, α1-AT, CNX32, and TAT and mRNA levels increased (P < 0.05) in differentiated bMSC at Day 28 of culture. Levels of CYP3A4 mRNA were higher (P < 0.05) in differentiated compared to control bMSC from Day 7 until Day 28 of culture. NANOG mRNA levels were lower (P < 0.05) in differentiated compared to control bMSC from Day 14 to Day 28 of culture. (*) Superscripts represent significant (P < 0.05) differences between treatments and sampling days. Bar scale: 500 μm.

Culture of bMSC in monolayer under hepatogenic conditions induced polygonal cell morphology and formation of cell aggregates at Day 28 of culture (Figure 2A). AFP protein was immunodetected in differentiated bMSC at Day 28 of culture (Figure 2B). At this stage, levels of *AFP*, *ALB*, *α1AT*, *CNX32*, *TAT* mRNA levels were up-regulated (P < 0.05) in differentiated bMSC (60.6-, 331.4-, 166.8-, 201.1-, 446.5-fold relative to Day 0, respectively) (Figure 2B). Levels of *CYP3A4* mRNA were higher (P < 0.05) in differentiated compared to control bMSC from Day 7 until Day 28 of culture (154.7- and 104.4-fold relative to Day 0 vs. 69- and 34-fold in untreated controls). In contrast, *NANOG* mRNA levels were lower (P < 0.05) in differentiated bMSC at Days 14, 24 and 21 of culture (1.2-. 0.86- and 0.97-fold relative to Day 0 vs. 8.6, 6.4- and 4.6-fold in untreated controls).

### Functional characterization of bMSC during hepatogenic differentiation

**Figure 3:**
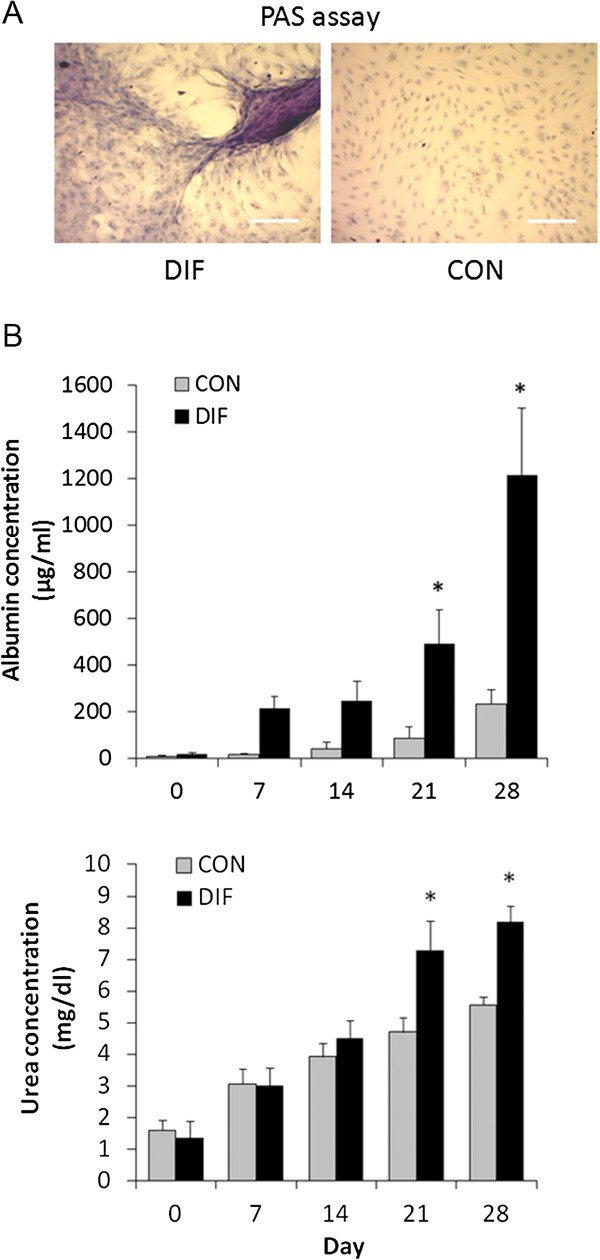
**Functional hepatocyte characterization of bMSC under hepatogenic conditions during a 28-Day *in vitro *culture period. (A)** More intense positive PAS staining was observed in differentiated (DIF) compared to control (CON) bMSC cultures. **(B)** Production of albumin increased (P < 0.05) in differentiated bMSC cultures at Days 21 and 28 of hepatogenic differentiation. Similarly, levels of urea increased (P < 0.05) in differentiated bMSC at Days 21 and 28 of hepatogenic differentiation. (*) Superscripts represent significant (P < 0.05) differences between treatments. Bar scale: 500 μm.

More intense PAS staining associated to stored glycogen was observed in differentiated compared to control bMSC at Day 28 of culture (Figure 3A). At Days 21 and 28 of differentiation, higher (P < 0.05) secretion of albumin (494.1 and 1213 μg/mL vs. 88.2 and 232.9 μg/mL in untreated controls) and urea concentration (7.3 and 8.2 mg/dL vs. 4.7 and 5.6 mg/dL in untreated controls) were detected in differentiated bMSC cultures (Figure 3B).

### Gene expression profile in bMSC during neurogenic differentiation

**Figure 4:**
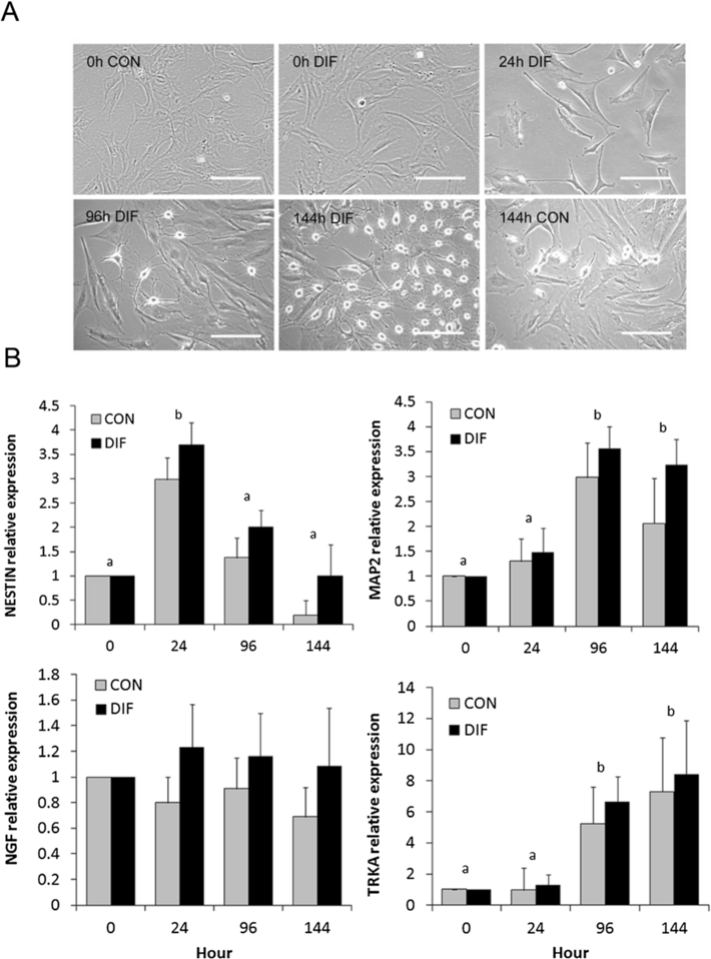
**Neural and multipotent gene expression profile in bMSC under differentiating conditions using neurogenic protocol 1. (A)** bMSC were isolated by plastic adherence from bovine fetal BM and cultured under neurogenic or control conditions. Culture of fetal bMSC under neurogenic conditions induced formation of cell body retraction and formation of multiple and complex extensions since 24 h of culture. **(B)** Analysis of Q-PCR detected mRNA levels of neural markers *NESTIN*, *MAP2*, *NGF* and *TRKA* in fetal bMSC cultured under control (CON) and differentiating (DIF) conditions. No differences (P > 0.05) in mRNA levels were detected between control and differentiated cultures. (a,b) Superscripts represent significant (P < 0.05) differences between sampling hour. Bar scale: 500 μm.

**Figure 5:**
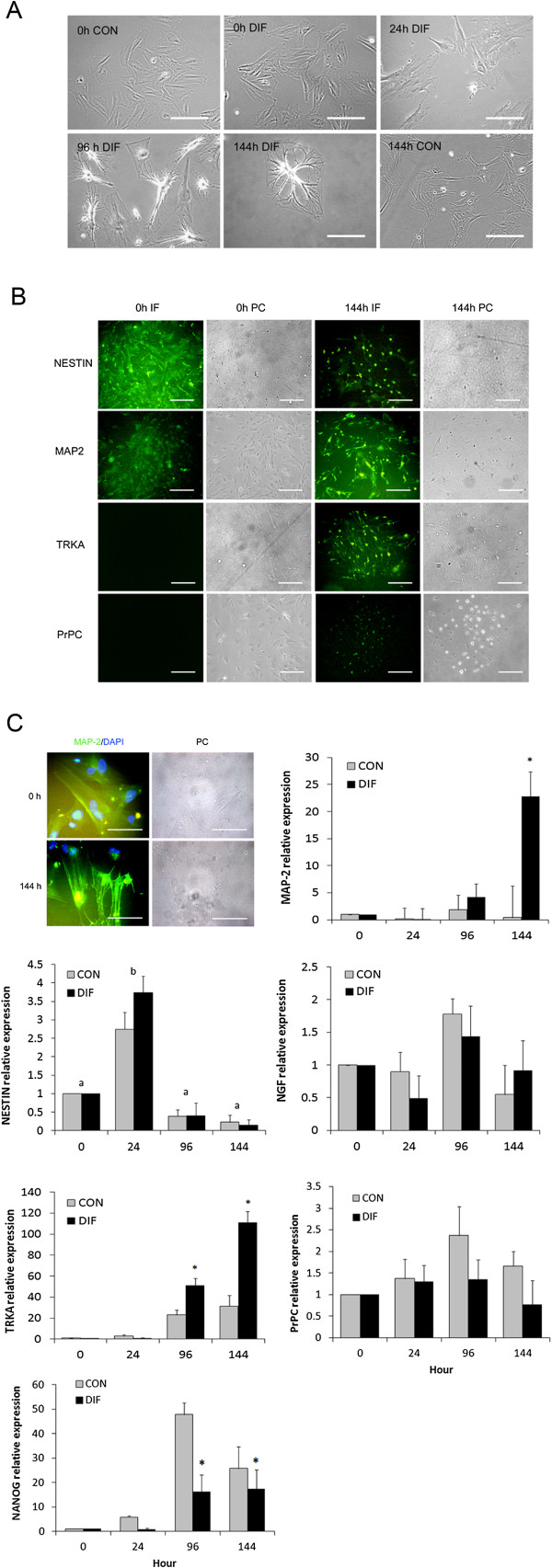
**Neural gene expression profile in bMSC under differentiating conditions using neurogenic protocol 2. (A)** Culture of fetal bMSC under neurogenic conditions induced formation of polygonal cell morphology and cell aggregates at Day 28 of culture. **(B)** At 24 h of culture, differentiated bMSC expressed both NESTIN and MAP2 proteins; whereas, at 144 h of culture also expressed TRKA and PrP^C^ proteins. **(C)** MAP2 protein was immunodetected in cell cytoplasm associated to the cytoskeleton. Levels of *MAP2* mRNA were up-regulated in differentiated (DIF) bMSC at 144 h of culture. In addition, these cells expressed higher (P < 0.05) levels of *TRKA* mRNA at 96 and 144 h of culture compared to controls. No differences in mRNA levels were detected for *NESTIN*, *NGF* and *PrP*^*C*^ between differentiated and control bMSC cultures. *NANOG* mRNA levels were lower (P < 0.05) in differentiated compared to control bMSC from 96 h to 144 h of culture. (*, a,b) Superscripts represent significant (P < 0.05) differences between treatments and sampling days, respectively. Bar scale: 500 μm.

Protocol 1 used for neurogenic differentiation induced formation of compact and spherical cell bodies with multiple extensions since 96 h of culture (Figure 4A). No significant differences in mRNA levels of neural markers *NESTIN*, *MAP2*, *NGF* and *TRKA* were detected between bMSC cultured under differentiating and control conditions (Figure 4B). Protocol 2 induced cell body retraction and formation of multiple and complex cell extensions in differentiated bMSC since 96 h of culture (Figure 5A). These cells expressed NESTIN and MAP2 proteins at 0 h of culture; whereas, at 144 h also expressed TRKA and PrP^C^ (Figure 5B and C). Differentiated bMSC expressed higher (P < 0.05) levels of *MAP2* mRNA (22.8-fold relative to Day 0 vs. 0.42-fold in untreated controls) at 144 h of culture (Figure 5C). Similarly, levels of *TRKA* mRNA were higher (P < 0.05) in differentiated bMSC at 96 h and 144 h of culture (51.4- and 111.2-fold relative to Day 0 vs. 23.1- and 31.3-fold in untreated controls). Conversely, differentiated bMSC expressed lower (P < 0.05) mRNA levels of *NANOG* at 96 h and 144 h of culture (16.3- and 17.4-fold relative to Day 0 vs. 47.9- and 25.8-fold in untreated controls). No significant differences were detected for *NESTIN*, *NGF* and *PrP*^*C*^ mRNA levels between treated and untreated bMSC; however, both cell populations expressed higher (P < 0.05) levels of *NESTIN* at 24 h of culture.

## Discussion

In the present study, bMSC were isolated from BM collected from abattoir-derived fetuses based on the capacity to adhere to plastic substrate under monolayer culture conditions. These cells expressed MSC specific markers CD29 and CD73 and lacked expression of hematopoietic surface markers CD34 and CD45. Little information is available regarding expression of MSC surface markers in bMSC. Analyses by flow cytometry in bMSC isolated from the Wharton’s jelly detected high proportion of cells positive for CD29, CD73, CD90 and CD105 and negative for CD34 and CD45 [11]. Using Q-PCR, we reported a similar MSC marker profile in bMSC isolated from BM of abattoir-derived bovine fetuses [12]. Recently, these results have been confirmed by immunofluorescence and RT-PCR in bMSC isolated from fetal BM [23]. Thus, *in vitro* characterization presented in this study and in previous reports including adherence to plastic substrate, expression of mesenchymal markers and differentiation into mesodermal lineages suggests that bMSC isolated from fetal BM by plastic adherence fulfill the minimal criteria for definition of MSC.

The 2-step sequential hepatogenic differentiation protocol used in our study included early (EGF), middle (HGF), and late (OSM, dexamethasone, ITS and DMSO) differentiation factors [24]. HGF is an endocrine or paracrine factor essential for liver development that promotes cell survival and regeneration [25]. OSM is a member of the interleukin-6 cytokine family that increases cell size of hepatocytes and induces cell differentiation and formation of bile canaliculi [26]. The capacity to induce hepatocyte functions, albumin secretion and cytochrome activity may be enhanced when OSM is combined with DMSO [27]. Dexamethasone, a synthetic glucocorticoid that induces hepatic gluconeogenesis, promotes the expression of hepatocyte phenotype through the suppression of cell division [28]. The hepatogenic differentiation potential of fetal bMSC was evaluated using quantitative and qualitative analyses. Spindle shape and polygonal cell morphology observed at Day 28 in differentiated bMSC was not typical hepatocyte-like morphology described in previous studies. However, during mesenchyme-to-hepatocyte differentiation, expression of specific transcription factors of early endoderm, bipotential cells, and finally hepatocytes were up-regulated. It is recognized that AFP and ALB are abundant proteins synthesized from early fetal to adult mature hepatocytes [29,30]. TAT and α1AT are liver-specific enzymes involved in degradation of tyrosine and inhibition of proteases, and are known to be late markers of the hepatocyte lineage [31,32]. CNX32 is a major gap junction protein which account for the 90% of connexin proteins in the liver [33]. Expression of these genes at the latest stage of differentiation indicates that prolonged exposure to hepatogenic factors is required in order to trigger the mesenchymal-to-hepatic transition. However, increasing levels of CYP3A4 mRNA Day 7 of culture suggest that activation of catalytic enzymes involved in the hepatic metabolism occurs at early stages of hepatogenic differentiation [34]. Moreover, production of albumin and urea at Days 21 and 28 of hepatogenic differentiation demonstrate the acquisition of hepatocyte metabolic activity in differentiated bMSC [35]. These results along with detection of glycogen storage indicate that bMSC underwent transdifferentiation into the endodermic hepatogenic lineage in agreement with the attainment of the hepatocyte gene expression profile.

In order to investigate the ectodermal differentiation potential, bMSC were induced toward neurogenic lineage using a previously reported BME-based protocol [17]. bMSC morphology including formation of compact cell bodies with multiple extensions along with expression of neural stem cell marker NESTIN and mature neuron markers MAP2, NGF and TRKA, initially suggested the acquisition of neural properties in treated bMSC. However, absence of neural marker increment in bMSC after treatment with a BME indicates that this simple chemical neuronal induction protocol was not able to induce neural differentiation. Previous studies have reported that BME-based protocols may induce neuronal phenotype in other cell types lacking any stem cell characteristics (fibroblasts, HEK 293 and PC-12 cells) [36,37]. This neuron-like appearance was associated to disruption of cytoskeleton after cell stress due to toxic chemicals rather than cell differentiation.

A second protocol based on combination of growth factor pretreatment and induction with previously reported factors was assessed in fetal bMSC for neurogenic differentiation [38]. EGF-bFGF pretreatment have been associated to initiation of MSC cycle exit and activation of neural/neuronal gene expression pattern [39]. Thereafter, culture in differentiation medium containing KCL, valproic acid, forskolin and hydrocortisone has showed to induce neuronal morphological and immunocytochemical changes in human and murine adipose-derived stem cells [38]. In our study, morphology changes in bMSC during culture were characterized by cell body retraction and formation of multiple and complex cell extensions that were consistent with neuronal differentiation. These cells expressed NESTIN and MAP2 at 24 h of culture, in addition with TRKA and PrP^C^ after 144 h of culture. The capacity of MSC to coexpress immature and mature neuronal or glial proteins has been previously associated to the plasticity to differentiate in various tissues [40,41]. In our study, immunodetection of NESTIN, MAP2, TRKA and PrP^C^ proteins, along with up-regulation of *MAP2* and *TRKA* mRNA in differentiated bMSC indicated that these cells were induced toward neural linage. However, a functional assay will be required before concluding that these cells have the potential to become neurons. Lack of significant differences in *NESTIN*, *NGF* and *PrP*^*C*^ mRNA levels between bMSC treatments were probably due to reduced level of expression of these genes. Constituently expression of NESTIN and MAP2 in MSC cultured without any previous induction, have been previously associated to the presence of a multidifferentiated state, in which MSC may retain a native potential for differentiation [42]. Previously, it have been reported that after *in utero* transplantation of human MSC in sheep or systemic infusion in baboons, MSC are found distributed in several tissues including gastrointestinal, kidney, liver, and skin [43,44]. These studies support the hypothesis [42] that subpopulations of MSC are in reality in a multidifferentiated state and participate *in vivo* in the turnover and replacement of a wide variety of tissues. Furthermore, these subpopulations may also explain the expression of lineage-specific markers in supposedly undifferentiated MSC populations. Although expression of NESTIN has been associated to early acquisition of neuronal lineage, expression in other cell type including myogenic, endothelial and hepatic suggest that this protein may not play a unique role in neurogenic differentiation [45]. Considering the proposed role of PrP^C^ in neural differentiation of embryonic stem cells (ESC) [46] and its participation in bovine spongiform encephalopathy (BSE) [47], transdifferentiation of MSC into neural cells could provide a valuable *in vitro* model for the study of prion diseases. Using co-localization analyses in the neuroepithelium of bovine fetuses and an *in vitro* differentiating ESC model, it has been shown that PrP^C^ influences the differentiation of NESTIN-expressing cell lineages, including neural lineages [46]. These data provides clues of the complex signaling pathways that mediate ESC neural differentiation; however, further *in vitro* differentiation experiments would be required in order to elucidate if this interactions are similar in MSC.

The expression of pluripotent markers OCT4 and NANOG was analyzed with the aim to evaluate the state of multipotency in bMSC during mesenchymal differentiation. OCT4 and NANOG regulate the maintenance of pluripotent state in embryos and derived cells in most mammalian species [48,49]. These transcription factors had been proposed to play a similar role in adult stem cells [50]. However, recent reports have indicated that NANOG expression is associated to adaptation to *in vitro* cell growth conditions in differentiating MSC [51,12]. In our study, the patterns of expression of OCT4 and NANOG in undifferentiated MSC support the potential participation of these factors during the multipotent state. Moreover, our data suggest that the multipotent capacity of bMSC is reduced according to the acquisition of the hepatogenic phenotype and functional activity.

## Conclusions

The expression patterns of lineage-specific markers and the production of functional metabolites demonstrate the acquisition of hepatocyte and neuronal profiles in bMSC after independent *in vitro* hepatogenic and neurogenic independent differentiations. The simplicity of isolation and the potential to differentiate into several cell types lays the foundation for BM MSC isolated from abattoir-derived bovine fetuses, as an alternative source of MSC for investigation of biology and eventual applications for regenerative therapy in veterinary medicine.

## Methods

All procedures have been approved by the Bioethical Committee of the National Commission for Scientific and Technology Research (Fondecyt).

### Isolation and culture of bMSC from fetal bone marrow

Bone marrow was aspirated from bovine fetuses (n = 10; 7–8 months of gestation) collected at a local abattoir. The marrow was drawn from femoral marrow cavity into syringes containing high glucose Dulbecco’s Modified Eagle Medium (DMEM, Gibco, Grand Islands, NY, USA) supplemented with 10% fetal bovine serum (FBS), 1000 U heparin, 100 U/mL penicillin and 100 μg/mL streptomycin. Bone marrow samples were washed twice with phosphate-buffered saline (PBS) and twice with DMEM. Then cells were plated in DMEM (high glucose) supplemented with 10% FBS, 100 U/mL penicillin, 100 μg/mL streptomycin and 0.25 μg/mL amphotericin B. Cells were incubated at 38°C in a humidified atmosphere containing 5% CO_2_. Non-adherent cells were removed by changing the culture medium after 4 days. Following the initial 4 days, the medium was changed every 2–3 days. After 4 to 5 passages, cells were gently harvested when 90% confluent using 0.25% trypsin in 0.1% EDTA and used for differentiation experiments.

### Hepatogenic differentiation

Induction of hepatogenic differentiation was performed using an adapted protocol from a previously published report [24]. Cells (3×10^3^/cm^2^) at passages 3 to 5 were plated in T-25 culture dishes either in control or differentiation medium. Control medium consisted of DMEM (low glucose) supplemented with 5% FBS, 100 U/mL penicillin, 100 μg/mL streptomycin and 0.25 μg/mL amphotericin B. bMSC were cultured in control medium for 1 week followed by culture in FBS-free control medium for three weeks. Differentiation was induced by treating bMSC with Step-1 differentiation medium consisting in 57.5% DMEM (low glucose) and 37.5% MCDB-201 (Sigma-Aldrich, St. Louis, MO, USA) supplemented with 1x ITS (BD Biosciences, San Jose, CA, USA), 1 nM dexamethasone, 0.1 mM ascorbic acid 2-phosphate (Sigma), 10 ng/mL rhEGF (BD Biosciences), 100 μg/mL streptomycin, 100 U/mL penicillin, 2.5 μg/mL amphotericin B and 5% FBS for 7 days. Thereafter, bMSC were treated with Step-2 differentiation medium consisting in Step-1 differentiation medium supplemented with 0.1% DMSO (Calbiochem, Darmstadt, Germany), 10 ng/mL rhHGF (Sigma) and 10 ng/mL rhOSM (Sigma) for 21 days. Medium change was performed every other day and cell samples were obtained from control and differentiation bMSC at Days 0, 7, 14 and 21 of culture and analyzed for *β-ACTIN*, *AFP*, *ALB*, *α1AT*, *CNX32*, *TAT*, *CYP3A4* and *NANOG* expression by Q-PCR.

### Neurogenic differentiation

Induction of neurogenic differentiation was performed using adapted protocols from previously published reports [17,38]. Cells (5×10^3^/cm^2^) at passages 3 to 5 were plated in T-25 culture dishes either in control or differentiation medium. Control medium consisted of DMEM (low glucose) supplemented with 100 U/mL penicillin, 100 μg/mL streptomycin and 0.25 μg/mL amphotericin B. In protocol 1, bMSC were cultured in a pre-induction medium consisting in DMEM (high glucose) supplemented with 20% FBS, 1 mM β-mercaptoethanol (BME, Sigma), 100 U/mL penicillin, 100 μg/mL streptomycin and 0.25 μg/mL amphotericin B. After 24 h, MSC were cultured in induction medium consisting in DMEM (high glucose) supplemented with 1 mM BME, 100 U/mL penicillin, 100 μg/mL streptomycin and 0.25 μg/mL amphotericin B. In protocol 2, MSC were cultured in a pre-induction medium consisting in DMEM (high glucose) supplemented with 20% FBS, 10 ng/mL bFGF (BD Biosciences), 20 ng/mL EGF (BD Biosciences), 100 U/mL penicillin, 100 μg/mL streptomycin and 0.25 μg/mL amphotericin B. After 24 h, bMSC were cultured in induction medium consisting in DMEM (high glucose) supplemented with 200 μM BME, 25 mM KCL, 2 mM valproic acid, 10 μM forskolin, 1% neural supplement (N1) (All from Sigma), 100 U/mL penicillin, 100 μg/mL streptomycin and 0.25 μg/mL amphotericin B. Cells were cultured for a total period of 6 days at 38°C under a humidified atmosphere containing 5% CO_2_. Medium change was performed every other day and cell samples were obtained from control and differentiation bMSC at 0, 24, 96 and 144 h of culture and analyzed for glyceraldehyde-3-phosphate dehydrogenase (*GAPDH*), *NESTIN*, *MAP2*, *NGF*, *TRKA*, *PrP*^*C*^ gene expression.

### Flow cytometry

The expression of CD29, CD34, CD45, CD73, OCT4 and NANOG was detected in bMSC using flow cytometry. Cells were removed from culture dishes using 5 mM EDTA for 10 min at 38°C. Then cells were permeabilized using a Foxp3 kit (eBioscience, San Diego, CA, USA) by incubation at room temperature for 5 min with shaking. Then cells were centrifuged at 2000 RPM for 5 min and then in 2% normal rabbit serum in PBS. Then bMSC were incubated in a 1:100 goat primary polyclonal antibody (CD34, CD73, OCT4 and NANOG; Santa Cruz Biotechnology, Santa Cruz, CA, USA) or rabbit primary monoclonal antibody (CD29 and CD45; Santa Cruz Biotechnology) for 30 min on ice with shaking. Then cells were washed with PBS and centrifuged 2000 RPM for 5 min. The pellet was resuspended in 1.5 mL of PBS and 1:100 donkey anti-goat or anti-mouse secondary antibodies (Santa Cruz Biotechnology) and incubated for 30 min on ice. After 3 washes on PBS, the pellet was resuspended on cytometry buffer and analyzed using a FACS Calibur flow cytometer (BD Bioscience) using a 488 nm (blue) and 633 nm (red) laser light. The signal was analyzed using a Cellquest program (BD Bioscience). The threshold for negative events was set on the first decade of fluorescence level. Negative procedural control corresponded to replacement of secondary antibody with non-immune serum.

### RNA extraction and cDNA synthesis

Cells were collected and immediately fixed in RLT buffer (Qiagen, Incorporated, Valencia, CA, USA). Total RNA was extracted using RNeasy Mini kit (Qiagen) following manufacturer’s recommendations. The concentration and purity of the RNA in each sample were determined using spectrophotometry (BioRad Laboratories, Hercules, CA, USA). Total RNA was eluted in 30–50 μL of RNase free water. Samples were subjected to RT-PCR using Brilliant II SYBR Green RT-PCR kit (Agilent). The reaction protocol consisted of incubation for 5 min at 25°C, 15 min at 42°C, 5 min at 95°C and hold at 4°C using a DNA engine PCR thermocycler (BioRad).

### Quantitative-PCR

Real-time PCR primers were designed using PrimerExpress software (Applied Biosystems Incorporated, Foster City, CA) (Table 1). Equivalence of amplification efficiencies among all primer-probe sets was confirmed using serial 3-fold dilutions of differentiated bMSC cDNA. Each RT-PCR reaction (25 μL) contained the following: 2X Brilliant II SYBR Green QPCR master mix (12.5 μL), diluted reference dye (0.375 μL), target forward primer (200 nM), target reverse primer (200 nM), cDNA synthesis reaction (2 μL) and nuclease-free PCR-grade water to adjust final volume. The PCR amplification was carried out in StepOne Real Time PCR System (Applied Biosystems). Thermal cycling conditions were 95°C for 10 min, followed by 40 repetitive cycles at 95°C for 30 sec and 60°C for 1 min. As a normalization control for RNA loading, parallel reactions in the same multiwell plate were performed using *GAPDH* or *β-ACTIN* as a target. Quantification of gene amplification was made following Q-PCR by determining the threshold cycle (C_T_) number for SYBR fluorescence within the geometric region of the semilog plot generated during PCR. Within this region of the amplification curve, each difference of one cycle is equivalent to a doubling of the amplified product of the PCR. The relative quantification of the target gene expression across treatment was evaluated using the comparative ΔΔC_T_ method. The C_T_ value was determined by subtracting the most stable endogenous gene C_T_ value (*β-ACTIN* hepatogenesis; *GAPDH*, neurogenesis) from the target C_T_ value of the sample. Calculation of ΔΔC_T_ involved using target gene expression on Day 0 (Sample with the highest CT value or lowest target expression) as an arbitrary constant to subtract from all other C_T_ sample values. Relative target mRNA expression was calculated as fold changes in relation to Day or Hour 0 sample and expressed as 2^-∆∆CT^ value.

**Table 1:** Sequence of primers used for Q-PCR analysis

**Gene**	**Sense**	**Antisense**	**Accession number**
**Housekeeping**			
GAPDH	5’ CCTTCATTGACCTTCACTACATGGTCTA	5’ TGGAAGATGGTGATGGCCTTTCCATTG	NM 001034034.2
βACTIN	5’ CGCACCACTGGCATTGTCAT	5’ TCCAAGGCGACGTAGCAGAG	K00622.1
**Hepatogenic**			
AFP	5’ CTTGTTGCTTACACAAAGAAGG	5’ ATGGAAGATGAACTTGTCATCG	NM 001034262.2
ALB	5’ TTTTCTCAGTATCTCCAGCAGT	5’ AGTAGGGATGTCTTCTAGCAAT	NM 180992.2
α1AT	5’ GCTGGGGTTCTCCAAGGAC	5’ GTTTGCTCATTCACGTGGAAGTC	NM 173882.2
CNX32	5’ ATGAACTGGACAGGTTTGTAC	5’ ATGTGTTGCTGGTGAGCCA	NM 174069.2
CYP3A4	5’ ATCATTGCTGTCTCCAACCTTCAC	5’ TGCTTCCCGCCTCAGATTTCTC	NM 001099367.1
TAT	5’ TTTGCTATGGAGCTTTGGCTGC	5’ AATGGTAGTGCAGCTCGCAGAA	NM 001034590.1
**Neurogenic**			
NESTIN	5’ CTGGAGCAGGAGAAACAAGG	5’ GAAAGGTTGGCACAGGTGTT	NM 001206591.1
MAP2	5’ CCACCTGAGATTAAGGATCA	5’ GGCTTACTTTGCTTCTCTGA	NM 001205807.1
NGF	5’ TCAACAGGACTCACAGGAGCAA	5’ ACCTCTCCCAGCACCATCAC	NM 001099362.1
TRKA	5’ CTGGGTGAGGGTGCCTTT	5’ CGCTCTCAGACACCTCCTTCAG	XM 002685965.3
PrP^C^	5’ CCAGAGACACAAATCCAACTTGAG	5’ AACCAGGATCCAACTGCCTATG	NM 001271626.1
**Pluripotency**			
NANOG	5’ GTGTTTGGTGAACTCTCCTG	5’ GGGAATTGAAATACTTGACAG	NM 001025344.1

### Immunofluorescence

Differentiated and control bMSC were fixed in a 4% paraformaldehyde (PAF) for 10 min and permeabilized with 0.1% Triton X-100 for 10 minutes. Cells were then rinsed twice in PBS and blocked in donkey serum (Sigma) for 30 min at RT. Cells were incubated over-night at 4°C with primary mouse monoclonal anti AFP or PrP^C^ antibody (1:50; Santa Cruz Biotechnology and Cayman Chemical, Ann Arbor, MI) or primary goat polyclonal anti NESTIN, MAP2, TRKA antibody (1:50; Santa Cruz Biotechnology) diluted in donkey serum. Then cells were rinsed three times with PBS and incubated with goat anti-mouse or anti-goat IgG conjugated to FITC (1:200 in donkey serum) for 45 minutes. Then cells were again rinsed three times in PBS and mounted under coverslips in a solution containing 4’, 6-diamidino-2-phenylindole (Santa Cruz Biotechnology). Samples were examined under epifluorescence and the results captured by digital photomicroscopy (Olympus, Tokyo, Japan).

### Periodic Acid-Schiff (PAS)

Glycogen storage was evaluated in differentiated and control bMSC using PAS staining. Culture dishes containing cells were fixed in 4% paraformaldehyde for 10 min. Cells were rinsed 3 times in deionized (d) H_2_O and treated with 0.5% periodic acid for 5 min at RT. Then cells were rinsed 3 times in dH_2_O, treated with Schiff’s reagent for 15 minutes, and rinsed 3 times in dH_2_O. Samples were counterstained with Mayer’s hematoxylin for 1 minute and rinsed in dH_2_O and assessed under light microscope.

### Urea assay

Urea concentrations were determined in media collected from control and differentiated bMSC cultures by colorimetric assay (Bioclin, Quibasa Quimica, Santa Branca, Brazil) following manufacturer’s recommendations and analyzed with spectrophotometer.

### Albumin assay

Albumin concentrations were determined in media collected from control and differentiation MSC cultures by Bromocresol green colorimetric assay (Biosystem, Barcelona, Spain) following manufacturer’s recommendations and analyzed with spectrophotometer.

### Data analysis

Values of gene expression from four different replicates were transferred to a spreadsheet and then analyzed using Infostat software. Data was normalized to logarithmic scale in base 10 for normality and mean values for each replicate were compared by one-way ANOVA. Gene expression values between days of culture and between treatments and controls were analyzed using Duncan’s multiple comparison test (P < 0.05).

## Abbreviations

bMSC: Bovine mesenchymal stem cell; CD73: Ecto-5′-nucleotidase; CD90: Thy-1; CD105: Endoglin; CD45: Protein tyrosine phosphatase; C: Receptor type; CD34: CD34 molecule; AFP: α-fetoprotein; ALB: Albumin; α1AT: Alpha1 antitrypsin; CNX32: Connexin 32; TAT: Tyrosine aminotransferase; CYP3A4: Cytochrome P450; NESTIN: Neuroepithelial stem cell intermediate filament; MAP2: Microtubule associated protein 2; NGF: Nerve growth factor; TRKA: Tropomyosin-related kinase A; PrPC: Cellular prion protein; bFGF: Bovine fibroblast growth factor; EGF: Epidermal growth factor; FBS: Fetal bovine serum; Q-PCR: Quantitative-polymerase chain reaction.

## Competing interests

The authors declare that they have no competing interests.

## Authors’ contributions

FD, VB and YC performed cell isolation and culture expansion, hepatogenic and neurogenic differentiation assays, statistical analyses and helped in the manuscript drafting. SV and LS participated in the gene expression and functional analyses. JP and MDR helped in the epifluorescence microscopy and in the manuscript drafting. OAP conceived and designed the study, analyzed the data and drafted the manuscript. All authors read and approved the final manuscript.
